# Editorial: Methods in biosensors and biomolecular electronics

**DOI:** 10.3389/fbioe.2023.1294221

**Published:** 2023-10-17

**Authors:** Tatiana Fiordelisio

**Affiliations:** ^1^ Laboratorio de Neuroendocrinología Comparada, Facultad de Ciencias, Universidad Nacional Autónoma de México, Mexico City, Mexico; ^2^ Laboratorio Nacional de Soluciones Biomiméticas para Diagnóstico y Terapia LaNSBioDyT, Facultad de Ciencias, Universidad Nacional Autónoma de México, Mexico City, Mexico

**Keywords:** biosensor, point-of-care, biological detector, immunosensors, genosensors, electrochemical biosensor, optical biosensor, diagnostic assays

Initially, biosensors were considered to be any analytical probe that provided a quantifiable signal in a biological medium, such as pH and oxygen electrodes. Today, we define them as analytical devices that can determine the presence of biological elements or biomolecules in a sample and are capable of converting a physical or chemical signal into an optically or electrically measurable signal.

**FIGURE 1:**
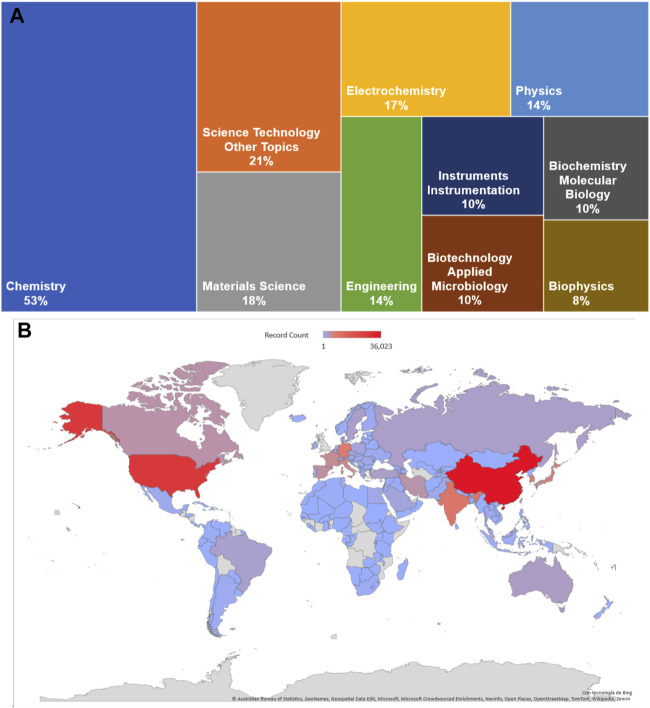
Topic search on biosensor or biomolecular electronics in Web of Science Core Research Topic (all editors 1900–2023). **(A)** Comparative graph of the percentage of developing areas in biosensors. **(B)** Heat map of the countries and its development on biosensors studies.

In 1962, Clark and Lyons developed the first electrochemical biosensor to measure glucose. Since then, biosensor development and popularity has grown substantially. The research areas with the highest presence are Chemistry (53.0%), Science Technology (21.4%), and Materials Science (17.5%). It is noteworthy that fields with great potential in their own right have a relatively low presence, such as Agriculture (0.5%), which is ranked 25th (Figure 1). The leading countries are the People’s Republic of China (26.7%) and the United States of America (20.0%).

Biosensors can be categorized into: qualitative, semi-quantitative or quantitative, covering a wide and dynamic range of information. Any biosensor must include three units specialized in detection, translation and processing. They are able to detect a wide range of biomarkers (nucleic acids, metabolites, hormones, proteins) and are therefore often used as diagnostic or prognostic tests for medical conditions. Molecular recognition of biomarkers by the detection unit depends on the affinity and specificity that results from thermodynamic equilibrium to form stable complexes. In this sense, innovation is needed to improve the affinity and stability of such bindings.

Depending on the characteristics of the biological detector, we have immunosensors, based on antigen-antibody recognition to measure biomolecules (as developed in this Research Topic by Liang et al. for the detection of 17β-estradiol) and genosensors, based on the detection of nucleic acids, which are very useful due to their high biocompatibility and thermal stability, (which can be used in many forms of biosensing, especially for viral infections). Their coupling to nanoparticles by Wang et al. used in the development of biosensors focused on infections such as *Candida* albicans is presented in this Research Topic.

In terms of the signal translation unit, both electrochemical and optical signals are considered. The electrochemical ones have high sensitivity and specificity, are low-cost and a very simple structure; they have also been miniaturized. Within these, there are those that measure conductometry, voltammetry, potentiometry and impedance. Other uses of electrical biosensors have been applied recently, such as the work done by Abadijoo et al. in developing electrical fields to prevent cytokine storms.

Optical translation biosensors have received a lot of attention given their usefulness in clinical diagnostics, drug measurement, food quality control, and even environmental monitoring. Most of them use the evanescent field to detect and measure molecules, they can use the surface plasmon wave where a change in refractive index caused by molecular interactions is detected, and there are also waveguide interferometers based on an optical resonator and fiber optics (Chen and Wang, 2020).

There has been a boom in the development of point-of-care (PoC) biosensors for diagnostics, which aim to meet the ideal ASSURED properties: Affordable, Sensitive, Specific, User-friendly, Rapid and Robust, Equipment-free and Delivered (Mabey et al., 2004; Drain et al., 2014). The absence of amplification of both the recognition and the signal is responsible for most of these systems and the possibility of miniaturization (Ortiz and Loeffelholz, 2023). Some of these aspects are proposed by Nelson-Mora in the article “New detection method of SARS-CoV-2 antibodies towards a point-of-care biosensor”. On the other hand, the search for new materials that allow miniaturized multiplex detection has been a major endeavor; the article in this Research Topic on hydrogel silk-based microarrays and molecular beacons for reagentless PoC diagnostics provides important advances in this field. While PoC biosensors have been developed for a variety of clinical diagnostic tests (Chen et al., 2020), they have also become popular in veterinary medicine because they can be used in the field and require only a small amount of sample (Stern and Camus, 2023). However, one limitation to their use is the need for improved signal transducers.

The COVID-19 pandemic greatly influenced such developments. The goal was to rapidly reduce the high transmission rate and mortality. However, detection limits and dynamic ranges are key Research Topic, as these analytical features affect clinical parameters such as sensitivity and specificity. As a result, even though most of them could be applied on a large scale, many developments had very low sensitivity, leading to false negatives. Having PoC ASSURED biosensors can make a difference in controlling a pandemic. During this time, many new biosensors for viral RNA detection were developed, including the use of CRISPR-Cas as optical biosensors (Morales-Moreno et al., 2023), lamp assays, aptamers, antigens, nanoparticles, and plasmon resonance (Mobed and Sepehri Shafigh, 2021), and even adaptations of standardized techniques to improve the sensitivity and speed of a test, as observed in the work of Mao et al. on asymmetric stem-loop-mediated isothermal amplification of nucleic acids for DNA diagnostic assays by simple.

Measuring environmental conditions, pathogens or pests affecting agricultural crops has been the focus of few PoCs. However, because of the agricultural and global ecological implications, it is critical to pay attention to these Research Topic. The development of this line of research and innovation should be motivated by concerns for environmental sustainability, water monitoring, air quality, food quality as well as disease diagnosis and tracking. In this Research Topic, Arena-Ortiz et al. developed DNA microarrays for the identification of etiological agents, as sensors of environmental wellbeing.

The main problems that arise from research and development of biosensors have to do with relying on large, expensive equipment and requiring highly trained personnel. As well as improvement of the signal translation and measurement system so that they can be low-cost, miniaturized devices that can be used by a person at home, in a modest clinic or in the field.

This Research Topic in the series aims to highlight the latest experimental techniques and methods used to investigate fundamental questions in biosensors and biomolecular electronic research, from methods in sensors for PoC diagnostics to those in protein electronics.
